# Supplementary material for: Transcriptional changes in Plasmodium falciparum upon conditional knock down of mitochondrial ribosomal proteins RSM22 and L23
Source: PLoS One. 2022 Oct 6;17(10):e0274993. doi: 10.1371/journal.pone.0274993 (PMC9536634; doi:10.1371/journal.pone.0274993)

**S1 Fig: PfRSM22 protein domain and amino acid sequence alignment.** (A) The green bars represent the length of each protein, while the brown and pink bars indicate the InterPro RSM22 family and methyltransferase domain regions within each protein, respectively (drawn to scale). The numbers represent the length of each protein in amino acids. Protein families and domains were identified via InterPro search: (<https://www.ebi.ac.uk/interpro/search/sequence>). RSM22-like proteins are shown with Uniprot identifier: *Bradyrhizobium japonicum* Q89SB0, *Plasmodium falciparum* Q8IJD1, *Toxoplasma gondii* S8GCN9, *Tetrahymena thermophila* Q22CT3, *Homo sapiens* P82650, *Caenorhabditis elegans* P91862, *Saccharomyces cerevisiae* P36056, *Arabidopsis thaliana* Q8GW63, *Trypanosoma brucei* Q385R2. (B) Protein sequence alignment of RSM22 orthologues from the organisms listed above. Intensity bars indicating the quality represent conservation of amino acids across listed organisms.

**(A)**

**(B)**


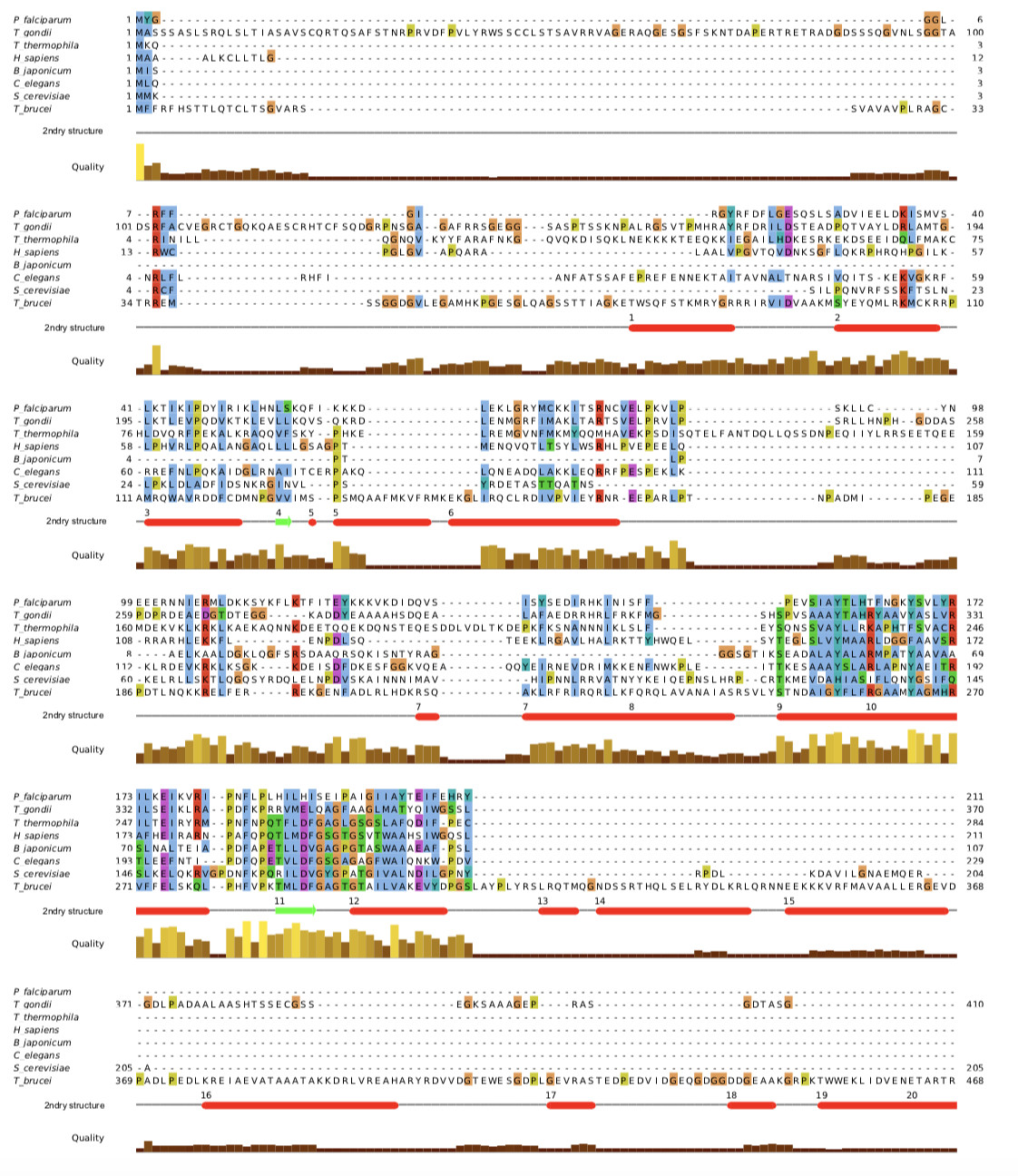


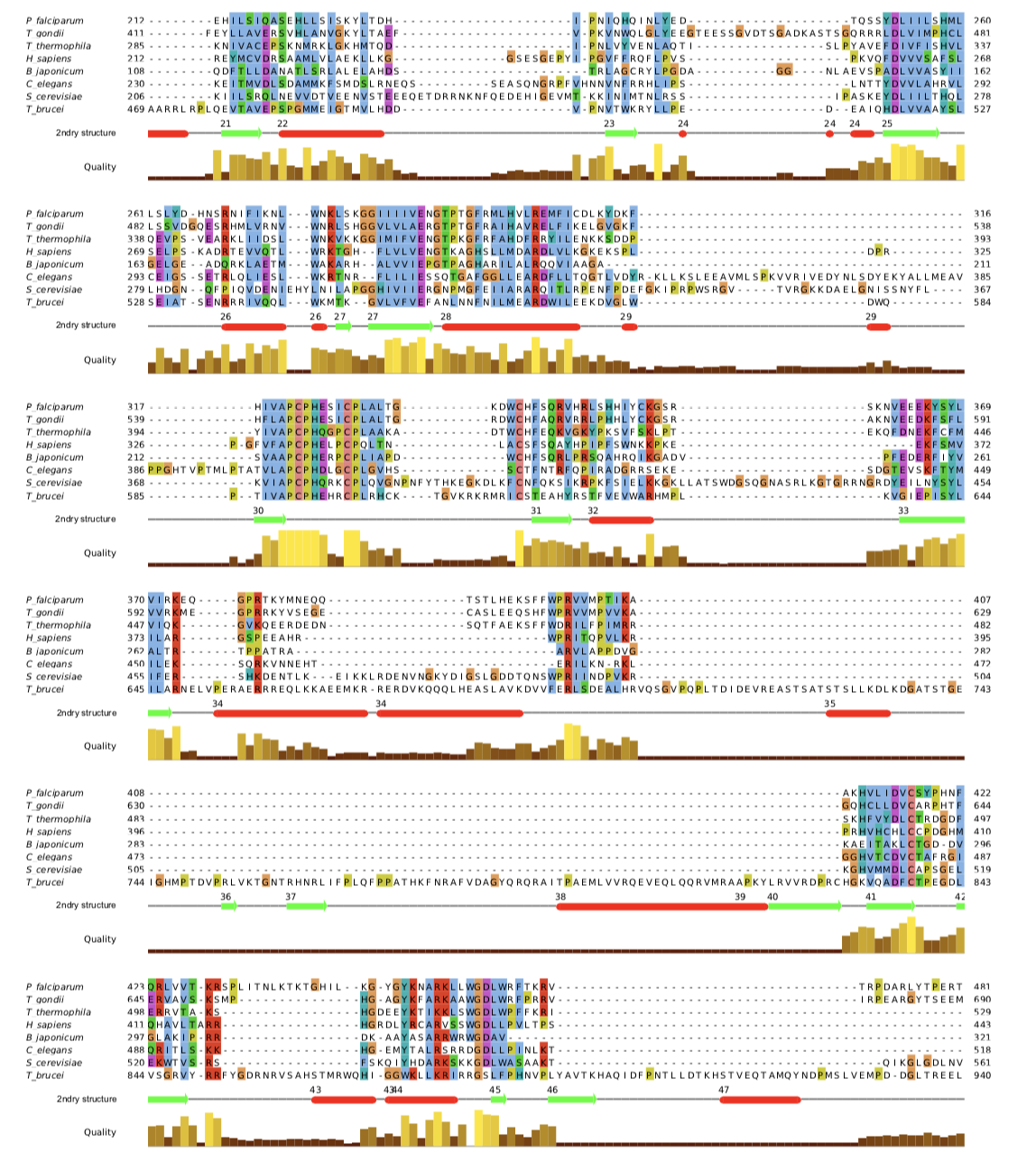


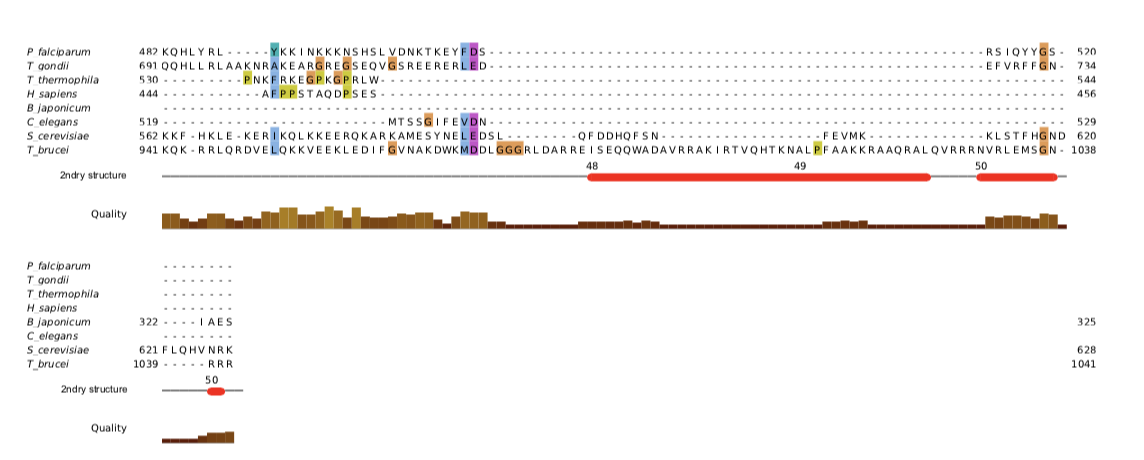

Supplement: S1 Fig — (DOCX) [file pone.0274993.s001.docx]
