# Supplementary material for: Transcriptional changes in Plasmodium falciparum upon conditional knock down of mitochondrial ribosomal proteins RSM22 and L23
Source: PLoS One. 2022 Oct 6;17(10):e0274993. doi: 10.1371/journal.pone.0274993 (PMC9536634; doi:10.1371/journal.pone.0274993)
Supplement: S2 Fig — (DOCX) [file pone.0274993.s002.docx]

**S2 Fig: PfMRPL23 protein domain and amino acid sequence alignment.** (A) Green and brown bars represent the length of each protein and uL23 protein family region within each protein, respectively. The numbers represent the length of each protein in amino acids. Protein families were identified via InterPro search(<https://www.ebi.ac.uk/interpro/search/sequence>). uL23 proteins are shown with Uniprot identifier: Escherichia coli P0ADZ0, *Rickettsia prowazekii* Q9ZCQ7. *Saccharomyces cerevisiae* P32387, *Homo sapiens* Q16540, *Plasmodium falciparum* Q8I532, *Toxoplasma gondii* A0A125YSK9, *Trypanosoma brucei* Q387G3, *Arabidopsis thaliana* Q9SMR5, *Tetrahymena thermophila* Q22EY1. (B) Protein sequence alignment of uL23 proteins from organisms listed above. Intensity bars indicating the quality represents conservation of amino acids across listed organisms.

**(A)**

**(B)**
