# Supplementary material for: Transcriptional changes in Plasmodium falciparum upon conditional knock down of mitochondrial ribosomal proteins RSM22 and L23
Source: PLoS One. 2022 Oct 6;17(10):e0274993. doi: 10.1371/journal.pone.0274993 (PMC9536634; doi:10.1371/journal.pone.0274993)
Supplement: S3 Fig — (DOCX) [file pone.0274993.s003.docx]

**S3 Fig: Endogenous tagging of PfRSM22 and PfMRPL23 using CRISPR/Cas9.** (A) A schematic of endogenous gene modification of PfRSM22 and PfMRPL23 using CRISPR/Cas9 mediated double crossover recombination. The pMG75-TetR-DOZI-8aptamer plasmid was linearized with EcoRV and transfected into WT D10 parasites together with corresponding circular gRNA plasmids. Via double crossover recombination, the homologous region (HR) segments of the gene locus of PfRSM22 or PfMRPL23 was inserted with a 3xHA tag and 8 aptamer repeats. Position of primers used to verify the parasite genotype in B is indicated. (B) Genotyping of D10-PfRSM22_3HA and D10-PfMRPL23_3HA parasite lines. DNA gel imaging of PCR products amplified from integrated or WT DNA, representing correct integration of 5’ HR and 3’ UTR at the expected gene loci. WT gene locus is intact in WT parasites.

(A)

**
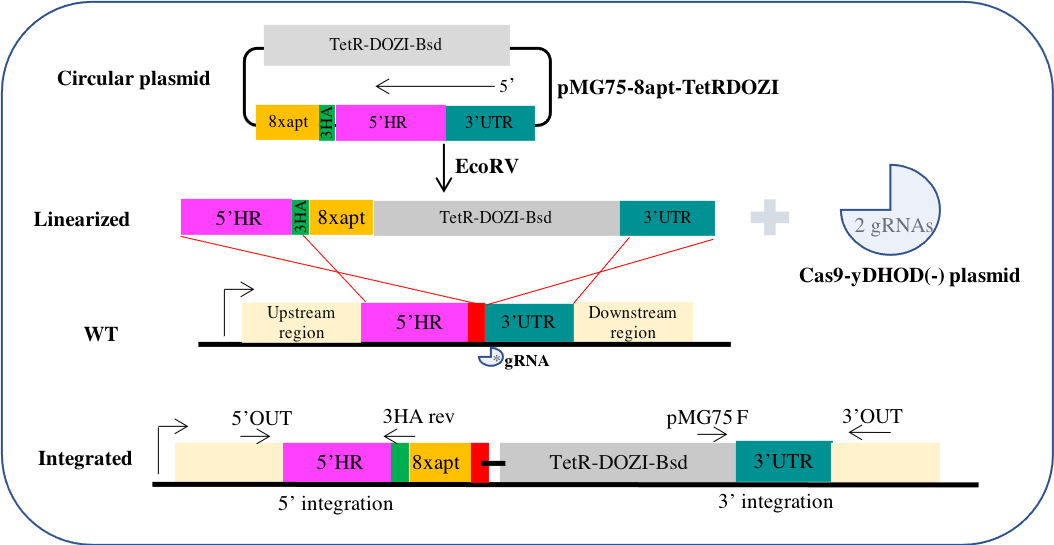
**

(B)
