# Supplementary material for: Transcriptional changes in Plasmodium falciparum upon conditional knock down of mitochondrial ribosomal proteins RSM22 and L23
Source: PLoS One. 2022 Oct 6;17(10):e0274993. doi: 10.1371/journal.pone.0274993 (PMC9536634; doi:10.1371/journal.pone.0274993)
Supplement: S4 Fig — (DOCX) [file pone.0274993.s004.docx]

**S4 Fig. RT-qPCR of representative SSU and LSU mt rRNA upon PfRSM22 and PfMRPL23 KD.** (A) Early and (B) late effects on the expression level of two mt-SSU and two mt-LSU rRNAs upon PfRSM22 KD (orange) and PfMRPL23 KD (blue) via RT- qPCR assay. Data shows mean + S.D of triplicates from n=2 independent experiments.

(B)

(A)
