# Supplementary material for: Transcriptional changes in Plasmodium falciparum upon conditional knock down of mitochondrial ribosomal proteins RSM22 and L23
Source: PLoS One. 2022 Oct 6;17(10):e0274993. doi: 10.1371/journal.pone.0274993 (PMC9536634; doi:10.1371/journal.pone.0274993)
Supplement: S5 Fig — (DOCX) [file pone.0274993.s005.docx]

**S5 Fig: Secondary structure of Pf mt rRNA fragments and their likely positions in the modeled SSU and LSU.** Secondary structures of SSU and LSU Pf mt rRNA fragments represented in light to dark grey color, subdivided into domain I, II, III, IV, V and VI. Transcripts uniquely downregulated upon PfRSM22 KD are represented in orange. No transcript was uniquely regulated upon PfMRPL23 KD. Transcripts differentially regulated upon KD of both PfRSM22 and PfMRPL23 are represented in teal. Purple color represents transcripts that were not detected in this study. The most updated *Plasmodium* mt rRNA map does not include position of 11 mt rRNA transcripts [3].

**
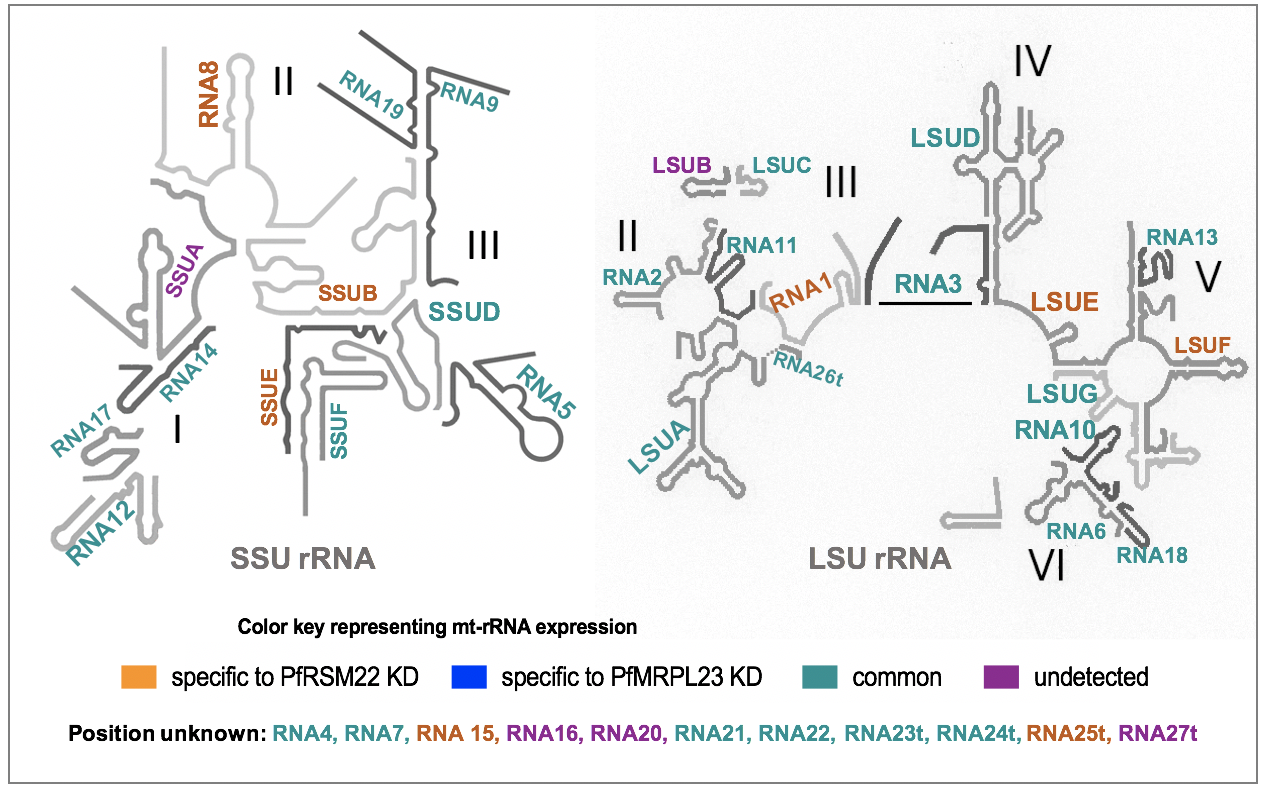
**
