# Supplementary material for: Transcriptional changes in Plasmodium falciparum upon conditional knock down of mitochondrial ribosomal proteins RSM22 and L23
Source: PLoS One. 2022 Oct 6;17(10):e0274993. doi: 10.1371/journal.pone.0274993 (PMC9536634; doi:10.1371/journal.pone.0274993)
Supplement: S6 Fig — (DOCX) [file pone.0274993.s006.docx]

**S6 Fig: Early and late effects of PfRSM22 and PfMRPL23 KD on apicoplast related transcripts.** (A) Heat map of differentially regulated transcripts common in the early phase of PfRSM22 and PfMRPL23 KD that are likely localized to the apicoplast. (B) Heat map of differentially regulated transcripts common in the late phase of PfRSM22 and PfMRPL23 KD that are likely localized to the apicoplast. Previously determined most updated list of apicoplast proteome was used for the analysis [4].

A)

Transcripts suggested to be localized to apicoplast

B)

PfRSM22 day6 off

PfMRPL23 day4 off

Transcripts suggested to be localized to apicoplast

PfMRPL23 day2 off

PfRSM22 day2 off
