# Supplementary material for: Transcriptional changes in Plasmodium falciparum upon conditional knock down of mitochondrial ribosomal proteins RSM22 and L23
Source: PLoS One. 2022 Oct 6;17(10):e0274993. doi: 10.1371/journal.pone.0274993 (PMC9536634; doi:10.1371/journal.pone.0274993)
Supplement: S8 Fig — (DOCX) [file pone.0274993.s008.docx]

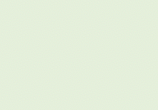


**PfRSM22 day6 off**

**PfMRPL23 day4 off**

**S8 Figure: Upregulated non-mitochondrial transcripts common between PfRSM22 and PfMRPL23 KDs in the late phase.** (A) Heat map of upregulated non mitochondrial 175 transcripts in common between PfRSM22 day6off and PfMRPL23 day4off. (B) List of GO term pathways of 175 transcripts generated on g:Profiler web server (<https://biit.cs.ut.ee/gprofiler/gost>)

A.

B.

B.

**
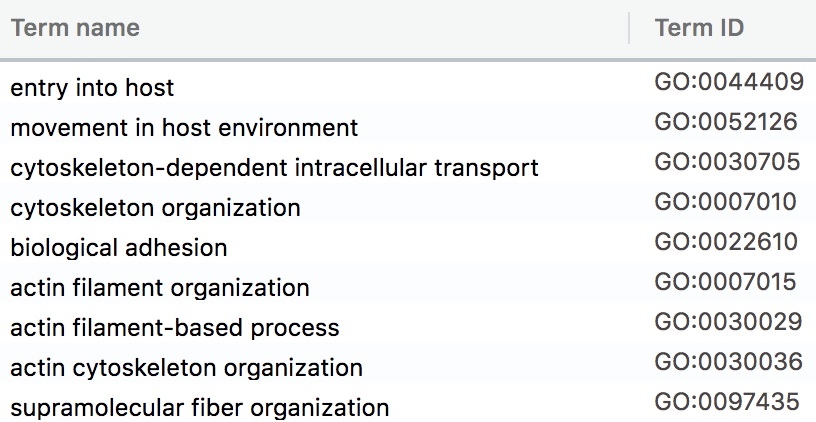
**
