# Supplementary material for: Transcriptional changes in Plasmodium falciparum upon conditional knock down of mitochondrial ribosomal proteins RSM22 and L23
Source: PLoS One. 2022 Oct 6;17(10):e0274993. doi: 10.1371/journal.pone.0274993 (PMC9536634; doi:10.1371/journal.pone.0274993)
Supplement: S1 Table — (DOCX) [file pone.0274993.s009.docx]

**S1 Table: List of primers and oligoes used in this study.**

|  | **Primers** | **Sequence** |
| --- | --- | --- |
| P1 | PFRSM22-5FOUT | AGATCACATACCAAATATAC |
| P2 | PFRSM22-5HRFWD | GGCCGCGGGATATCTCCGGAGTAGAGAATGGTACACCCACAG |
| P3 | PFRSM22-5HRREV | AAAATGTTTATCAAACCGGGGGTAACCTGTGATCCATAGTATTGAATTGATCTAGAATCGAAATATTCCTTAGTCTTGTT |
| P4 | PFRSM22-3UTRFWD | ATGGCCCCTTTCCGGGCGCGCCTTAAGGAATAACACAAGAGGTTATAGTTTA |
| P5 | PFRSM22-3UTRREV | TCCGGAGATATCCCGCGGCCTATTTGATGAGTGCATTATCC |
| P6 | PFRSM22-3FOUT | TAAAATAAGTGTGTGGTGCT |
| P7 | PFRSM22_gRNA1 | CATATTAAGTATATAATATTGTCACGTTCCATACAGTATTAGTTTCAGAGCTATGCTGGA |
| P8 | PFRSM22_gRNA1_N21 | GTCACGTTCCATACAGTATTA |
| P9 | PFRSM22_gRNA2 | CATATTAAGTATATAATATTATATGTGTAGCATATTTCTTGTTTCAGAGCTATGCTGGAA |
| P10 | PFRSM22_gRNA2_N20 | ATATGTGTAGCATATTTCTT |
| P11 | PMG75seqF | CTTTAAATTCATGCAAAAATTTAC |
| P12 | BBHA REV | TGGGCCCGAATTCTCATCATTGTGC |
| P13 | PFMRPL23-5FOUT | GAGCTTTGTTCGATATAC |
| P14 | PFMRPL23-5HRFWD | CTGGTTACCTAGATATCAACCGCGGATATGAACATGAAGTACCGGATAG |
| P15 | PFMRPL23-5HRREV | ATGTCGACCTGCAGTGAAGGTGGTTTCCACTTTGAAGAAGATGACTTCTTGATATTTTTATTTTCACTTGAATATTCAATTGTTTC |
| P16 | PFMRPL23-3HRFWD | ATCCGCGGTCTTAAGGCTTATTTTGTACATATTAG |
| P17 | PFMRPL23-3HRREV | CTGGTTACCATCAGAATTAAATATATACACATTC |
| P18 | PFMRPL23-3FOUT | CGCACATTCTAGTTTGATATTTTG |
| P19 | PFMRPL23_gRNA1 | CATATTAAGTATATAATATTGAAAAATCGTCAAGCAGTAAAGTTTTAGAGCTAGAAATAGC |
| P20 | PFMRPL23_gRNA1_N21 | AAAAATCGTCAAGCAGTAAAG |
| P21 | PFMRPL23_gRNA2 | CATATTAAGTATATAATATTGATTTTTAATTATGTACTATAGTTTTAGAGCTAGAAATAGC |
| P22 | PFMRPL23_gRNA2_N20 | ATTTTTAATTATGTACTATA |
| P23 | N20 CHECK REV | ATATGAATTACAAATATTGCATAAAGA |
|  |  |  |
|  | **RT-qPCR primers** | **Sequence** |
|  | mt rRNA 5 F | ATAGTTACCATAGCTGTAGATG |
|  | mt rRNA 5 R | GTTTTTGGCGGCTGAGCATGT |
|  | mt rRNA 8 F | CTCTACAAAGTTGAACATAGGCTGAGTC |
|  | mt rRNA 8 R | AACTTCTTATAAATGGAAGCGCCGG |
|  | mt rRNA 10 F | TATGTCCTGTTTCAAATATATAT |
|  | mt rRNA 10 R | TTTGATAGCGGTTAACCTTTCC |
|  | mt LSUA F | TTATAGCCATGTCTCCATGAACTAT |
|  | mt LSUA R | ATGATATATCTTCCAAATAGA |
|  | PfGAPDH F | TCCTTGGGGAAAATGCCAAGT |
|  | PfGAPDH R | TGGGGTGTCATCCTTTGGTG |
