# Supplementary material for: Transcriptional changes in Plasmodium falciparum upon conditional knock down of mitochondrial ribosomal proteins RSM22 and L23
Source: PLoS One. 2022 Oct 6;17(10):e0274993. doi: 10.1371/journal.pone.0274993 (PMC9536634; doi:10.1371/journal.pone.0274993)
Supplement: S1 File — (DOCX) [file pone.0274993.s014.docx]

**Supporting Information**

**Transcriptional changes in *Plasmodium falciparum* upon conditional knock down of mitochondrial ribosomal proteins RSMS22 and L23**

Swati Dass^1^, Michael W. Mather^1^, Joanne M. Morrisey^1^, Liqin Ling^1, #a^, Akhil B. Vaidya^1^, Hangjun Ke^1^

^1^Center for Molecular Parasitology, Department of Microbiology and Immunology, Drexel University College of 6 Medicine, Philadelphia, Pennsylvania, USA

^1, #a^Current address: Department of Laboratory Medicine, West China Hospital, Sichuan University, Chengdu, China.

***** Corresponding author

E-mail*:* [hk84@drexel.edu](mailto:hk84@drexel.edu)

1. Plasmid construction for knocking down PfRSM22 and PfMRPL23.
2. **S1 Fig.** PfRSM22 protein domain and amino acid sequence alignment.
3. **S2 Fig.** PfMRPL23 protein domain and amino acid sequence alignment.
4. **S3 Fig.** Endogenous tagging of PfRSM22 and PfMRPL23 using CRISPR/Cas9.
5. **S4 Fig.** RT-qPCR of representative SSU and LSU mt rRNA upon protein KD.
6. **S5 Fig.** Secondary structure of Pf mt rRNA fragments and their likely positions in the modeled SSU and LSU.
7. **S6 Fig.** Early and late effects of PfRSM22 and PfMRPL23 KD on apicoplast related transcripts.
8. **S7 Fig.** Downregulated non-mitochondrial transcripts common between PfRSM22 and PfMRPL23 KDs in the late phase.
9. **S8 Fig.** Upregulated non-mitochondrial transcripts common between PfRSM22 and PfMRPL23 KDs in the late phase.
10. **S1 Table.** List of primers and oligoes used in this study.
11. **S2 Table.** Read count files of the PfRSM22_3HA line (aTc ON and day 2, day4, day 6 aTc OFF) and the PfMRPL23_3HA (aTc ON and day 2, day 4 aTc OFF).
12. **S3 Table.** KEGG and Gene ontology (GO) term list of significantly regulated genes upon KD of PfRSM22 and PfMRPL23.
13. **S4 Table.** List of significantly up/downregulated genes, mitochondrial related genes, and genes in the Venn diagrams.
14. **S5 Table.** RNA sequencing read depth.
15. **Plasmid construction for knocking down PfRSM22 and PfMRPL23**

All primers and oligos were purchased from Eurofins Genomics or Genewiz LLC (S1 Table). DNA fragments were amplified using high fidelity DNA polymerases (New England Biolabs®, Inc) and confirmed by sequencing (Genewiz LLC). Transformation of pMG75 related plasmids was performed using Stable competent *E. coli* cells (New England Biolabs®, Inc) and bacteria were grown at 30°C to maintain the stability of 8X aptamer repeats. Transformation of Cas9 related plasmids was performed using DH5-alpha electrocompetent *E. coli* cells and bacteria were grown at 37°C.

The pMG75 vector [1] was used to modify the genomic locus of PfRSM22 (Pf3D7_1027200). Briefly, the original pMG75noP-ATP4-8apt-3HA plasmid was linearized by *Afl*II and *BstE*II*,* to remove the ATP4 inserts. PfRSM22 5’HR region was amplified from wild type *P. falciparum* (WT) genomic DNA using primers (P2+P3) giving a PCR product of 868 bp. The 3’HR region was downstream of the stop codon and was amplified using primers (P4 + P5) giving a PCR product of 601 bp in length. The three pieces including the linearized vector, PfRSM22 5’HR and PfRSM22 3’HR were joined together using NEB HiFi DNA assembly master mix. The gRNAs of PfRSM22 were present at the end of 5’HR, where Cas9 would introduce a cut to create the expected modification in the transgenic parasites. To avoid repetitive cutting, synonymous mutations were introduced in the reverse primer of the 5’HR (P3) within the gRNA region. The modified pMG75 vector was sequenced using primers P11 and P12 to verify the 3’HR and 5’HR inserts, respectively.

The PfMRPL23 5’HR and 3’HR were cloned into apMG75 derivative one after the other. Briefly, the pMG75 vector containing mt-DNA polymerase I (Pf3D7_0625300) was digested with BstEII and SacII to remove mt-DNA pol 3’ HR. PfMRPL23 3’ HR was amplified from WT parasite DNA using 3’HR primers flanked by BstEII at its reverse primer and SacII at its forward primer (P16 +P17). The amplified 3’HR (1000 bp) was digested with SacII and BstEII and inserted into the digested pMG75 vector. Presence of the PfMRPL23 3’HR and absence of the mt-DNA pol 3’HR was confirmed by PCR amplification followed by sequencing analysis. The pMG75 vector carrying PfMRPL23 3’HR was digested with SacII and SalI to remove mtDNA pol 5’HR. PfMRPL23 5’ HR was amplified using primers P14+P15. The amplified product was 853 bp in length and was digested with SacII and SalI and inserted into the pMG75 vector carrying PfMRPL23 3’HR, resulting in the final plasmid pMG75-PfMRPL23-3HA-8apt. The modified pMG75 vector was sequenced using primers P11 and P12 to the verify 3’HR and 5’HR insert sequences, respectively.

gRNAs were designed using Eukaryotic Pathogen CRISPR guide RNA Design Tool (<http://grna.ctegd.uga.edu/>). High efficiency score gRNAs having no off-target matches were selected and PfRSM22 gRNAs (P7 and P9) were cloned in a No flag (NF)-Cas9-yDHOD (-) plasmid construct. Briefly, NF-Cas9-yDHOD (-) vector [1] was digested with EcoRI and joined with oligos P7 and P9 individually by NEB HiFiDNA assembly. Correct infusion of PfRSM22 gRNA1-NF-Cas9-yDHOD (-) was confirmed by PCR using primers P8+P23 whereas PfRSM22 gRNA2 insertion was confirmed by PCR using P10+P23. The inserted gRNAs were further confirmed by sequencing analysis. PfMRPL23 gRNAs were cloned into the M-Cas9-yDHOD (-) vector which still had a flag tag present in Cas9 [2]. The M-Cas9-yDHOD(-) vector digested with EcoRI was joined with oligos P19 and P21 individually by NEB HiFiDNA assembly. Fusion of gRNAs into M-Cas9-yDHOD was verified by PCR using primers P20+P23 (gRNA1) or P22+P23 (gRNA2) and by sequencing.

1. **S1 Fig: PfRSM22 protein domain and amino acid sequence alignment.** (A) The green bars represent the length of each protein, while the brown and pink bars indicate the InterPro RSM22 family and methyltransferase domain regions within each protein, respectively (drawn to scale). The numbers represent the length of each protein in amino acids. Protein families and domains were identified via InterPro search: (<https://www.ebi.ac.uk/interpro/search/sequence>). RSM22-like proteins are shown with Uniprot identifier: *Bradyrhizobium japonicum* Q89SB0, *Plasmodium falciparum* Q8IJD1, *Toxoplasma gondii* S8GCN9, *Tetrahymena thermophila* Q22CT3, *Homo sapiens* P82650, *Caenorhabditis elegans* P91862, *Saccharomyces cerevisiae* P36056, *Arabidopsis thaliana* Q8GW63, *Trypanosoma brucei* Q385R2. (B) Protein sequence alignment of RSM22 orthologues from the organisms listed above. Intensity bars indicating the quality represent conservation of amino acids across listed organisms.

**(A)**

**(B)**


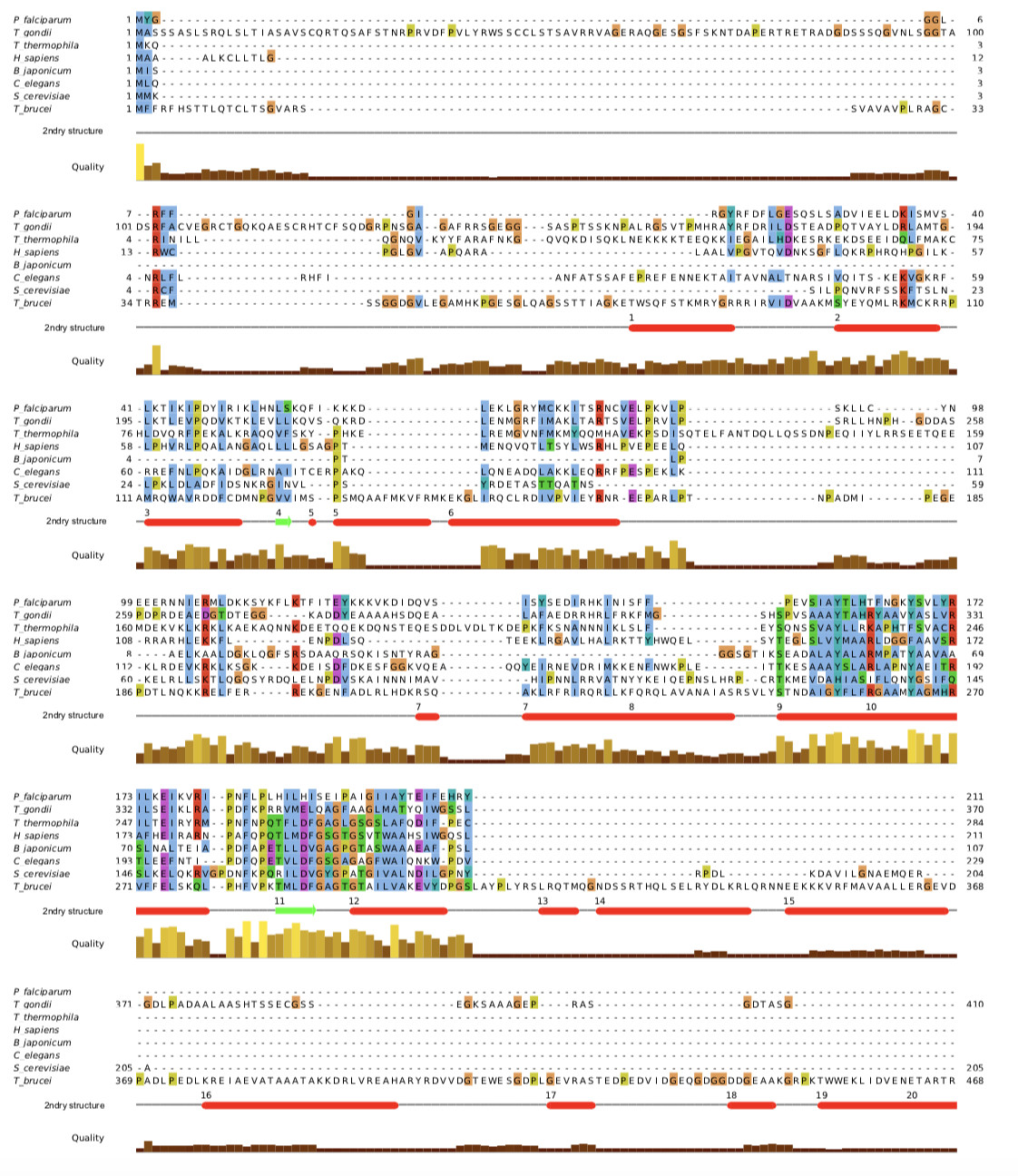


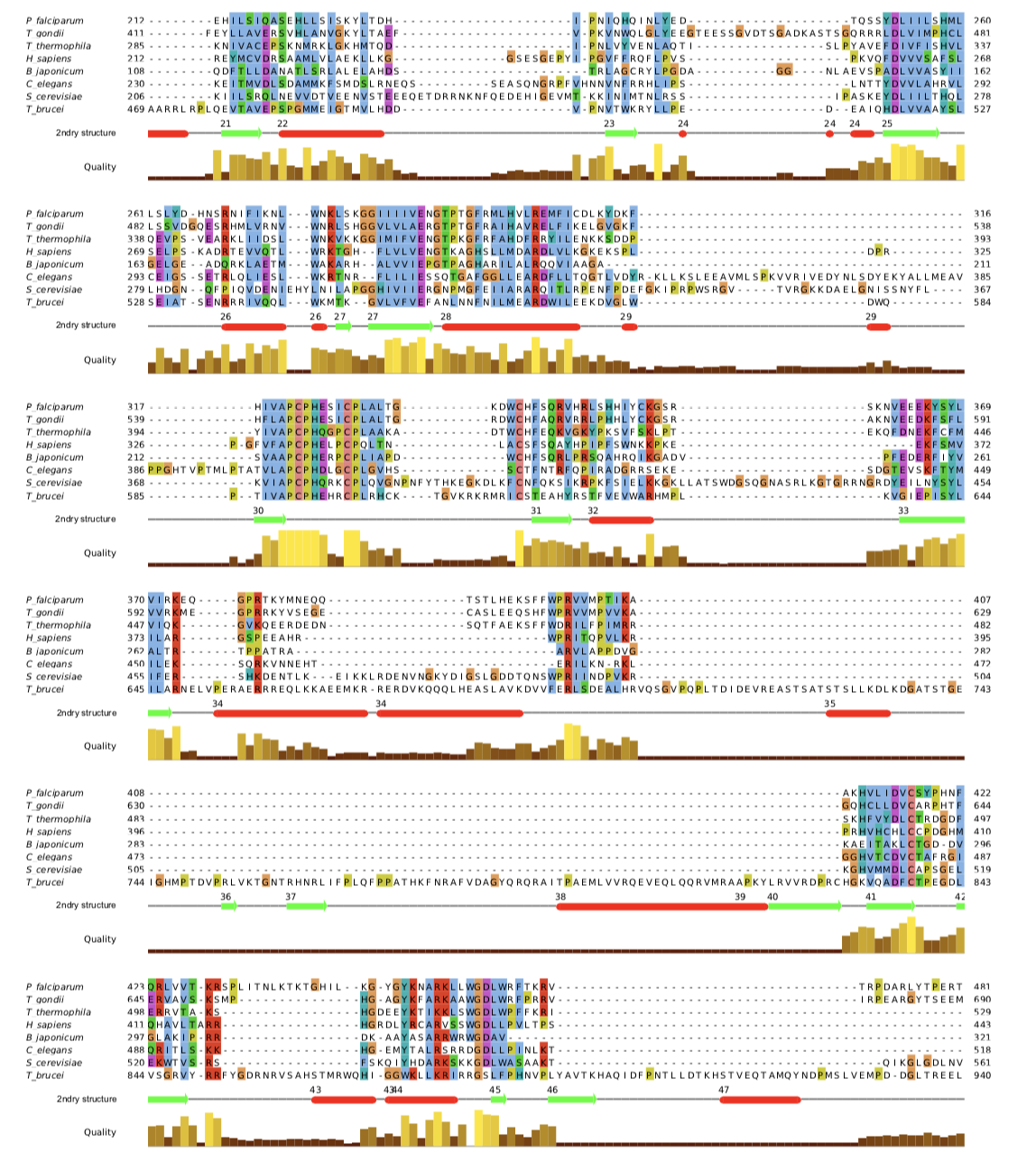


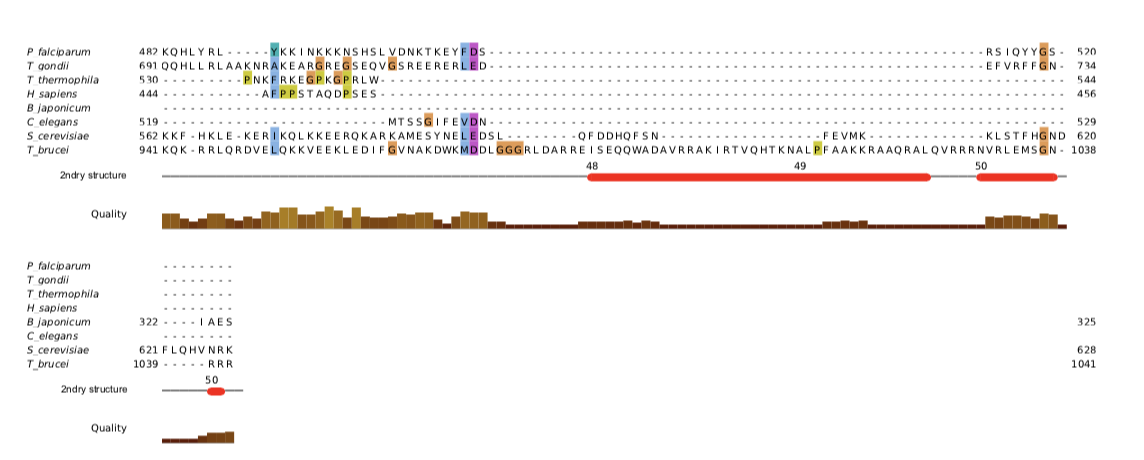


1. **S2 Fig: PfMRPL23 protein domain and amino acid sequence alignment.** (A) Green and brown bars represent the length of each protein and uL23 protein family region within each protein, respectively. The numbers represent the length of each protein in amino acids. Protein families were identified via InterPro search(<https://www.ebi.ac.uk/interpro/search/sequence>). uL23 proteins are shown with Uniprot identifier: Escherichia coli P0ADZ0, *Rickettsia prowazekii* Q9ZCQ7. *Saccharomyces cerevisiae* P32387, *Homo sapiens* Q16540, *Plasmodium falciparum* Q8I532, *Toxoplasma gondii* A0A125YSK9, *Trypanosoma brucei* Q387G3, *Arabidopsis thaliana* Q9SMR5, *Tetrahymena thermophila* Q22EY1. (B) Protein sequence alignment of uL23 proteins from organisms listed above. Intensity bars indicating the quality represents conservation of amino acids across listed organisms.

**(A)**

**(B)**

**4. S3 Fig: Endogenous tagging of PfRSM22 and PfMRPL23 using CRISPR/Cas9.** (A) A schematic of endogenous gene modification of PfRSM22 and PfMRPL23 using CRISPR/Cas9 mediated double crossover recombination. The pMG75-TetR-DOZI-8aptamer plasmid was linearized with EcoRV and transfected into WT D10 parasites together with corresponding circular gRNA plasmids. Via double crossover recombination, the homologous region (HR) segments of the gene locus of PfRSM22 or PfMRPL23 was inserted with a 3xHA tag and 8 aptamer repeats. Position of primers used to verify the parasite genotype in B is indicated. (B) Genotyping of D10-PfRSM22_3HA and D10-PfMRPL23_3HA parasite lines. DNA gel imaging of PCR products amplified from integrated or WT DNA, representing correct integration of 5’ HR and 3’ UTR at the expected gene loci. WT gene locus is intact in WT parasites.

(A)

**
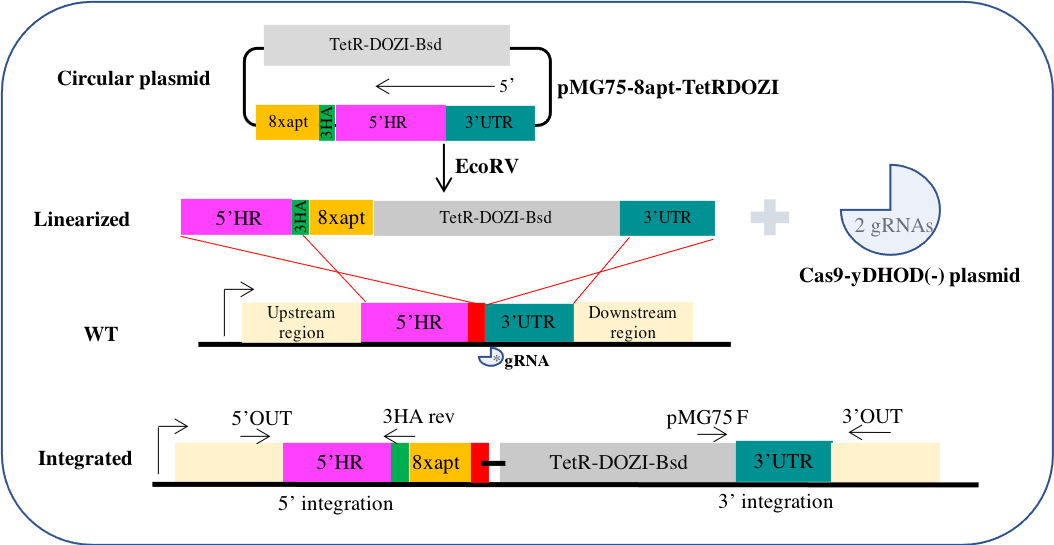
**

(B)

1. **S4 Fig. RT-qPCR of representative SSU and LSU mt rRNA upon PfRSM22 and PfMRPL23 KD.** (A) Early and (B) late effects on the expression level of two mt-SSU and two mt-LSU rRNAs upon PfRSM22 KD (orange) and PfMRPL23 KD (blue) via RT- qPCR assay. Data shows mean + S.D of triplicates from n=2 independent experiments.

(B)

(A)

1. **S5 Fig: Secondary structure of Pf mt rRNA fragments and their likely positions in the modeled SSU and LSU.** Secondary structures of SSU and LSU Pf mt rRNA fragments represented in light to dark grey color, subdivided into domain I, II, III, IV, V and VI. Transcripts uniquely downregulated upon PfRSM22 KD are represented in orange. No transcript was uniquely regulated upon PfMRPL23 KD. Transcripts differentially regulated upon KD of both PfRSM22 and PfMRPL23 are represented in teal. Purple color represents transcripts that were not detected in this study. The most updated *Plasmodium* mt rRNA map does not include position of 11 mt rRNA transcripts [3].

**
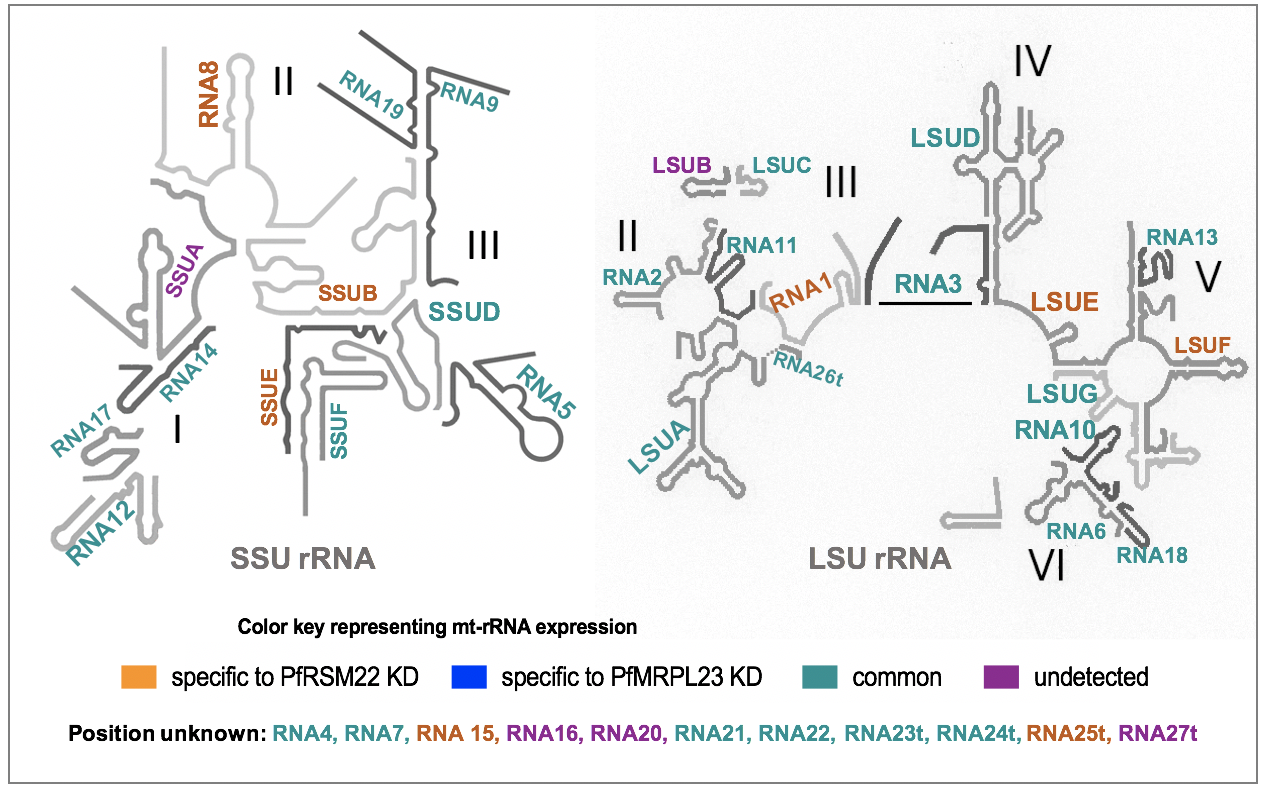
**

1. **S6 Fig: Early and late effects of PfRSM22 and PfMRPL23 KD on apicoplast related transcripts.** (A) Heat map of differentially regulated transcripts common in the early phase of PfRSM22 and PfMRPL23 KD that are likely localized to the apicoplast. (B) Heat map of differentially regulated transcripts common in the late phase of PfRSM22 and PfMRPL23 KD that are likely localized to the apicoplast. Previously determined most updated list of apicoplast proteome was used for the analysis [4].

A)

Transcripts suggested to be localized to apicoplast

B)

PfRSM22 day6 off

PfMRPL23 day4 off

Transcripts suggested to be localized to apicoplast

PfMRPL23 day2 off

PfRSM22 day2 off

**8. S7 Fig: Downregulated non-mitochondrial transcripts common between PfRSM22 and PfMRPL23 KDs in the late phase.** (A) Heat map of downregulated non mitochondrial 118 transcripts in common between PfRSM22 day6off and PfMRPL23 day4off. (B) List of GO term pathways of 118 transcripts generated on g:Profiler web server (<https://biit.cs.ut.ee/gprofiler/gost>)

A.

A)


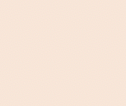


**PfRSM22 day6 off**

**PfMRPL23 day4 off**

B.

**
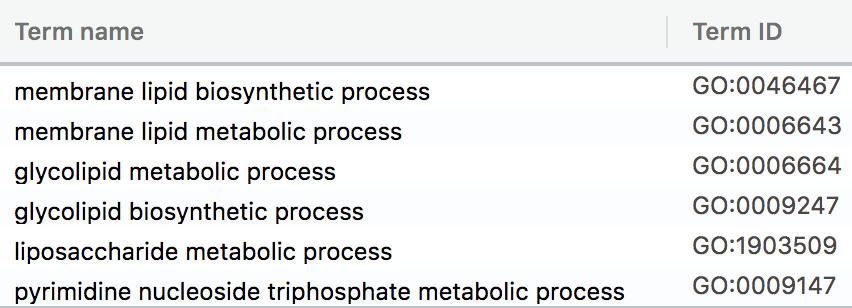
**


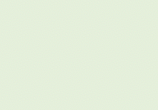


**PfRSM22 day6 off**

**PfMRPL23 day4 off**

**9. S8 Figure: Upregulated non-mitochondrial transcripts common between PfRSM22 and PfMRPL23 KDs in the late phase.** (A) Heat map of upregulated non mitochondrial 175 transcripts in common between PfRSM22 day6off and PfMRPL23 day4off. (B) List of GO term pathways of 175 transcripts generated on g:Profiler web server (<https://biit.cs.ut.ee/gprofiler/gost>)

A.

B.

B.

**
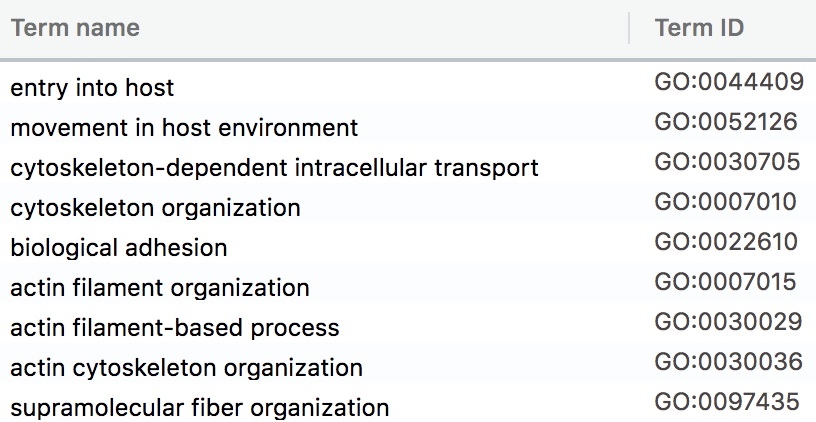
**

**4. S1 Table: List of primers and oligoes used in this study.**

|  | **Primers** | **Sequence** |
| --- | --- | --- |
| P1 | PFRSM22-5FOUT | AGATCACATACCAAATATAC |
| P2 | PFRSM22-5HRFWD | GGCCGCGGGATATCTCCGGAGTAGAGAATGGTACACCCACAG |
| P3 | PFRSM22-5HRREV | AAAATGTTTATCAAACCGGGGGTAACCTGTGATCCATAGTATTGAATTGATCTAGAATCGAAATATTCCTTAGTCTTGTT |
| P4 | PFRSM22-3UTRFWD | ATGGCCCCTTTCCGGGCGCGCCTTAAGGAATAACACAAGAGGTTATAGTTTA |
| P5 | PFRSM22-3UTRREV | TCCGGAGATATCCCGCGGCCTATTTGATGAGTGCATTATCC |
| P6 | PFRSM22-3FOUT | TAAAATAAGTGTGTGGTGCT |
| P7 | PFRSM22_gRNA1 | CATATTAAGTATATAATATTGTCACGTTCCATACAGTATTAGTTTCAGAGCTATGCTGGA |
| P8 | PFRSM22_gRNA1_N21 | GTCACGTTCCATACAGTATTA |
| P9 | PFRSM22_gRNA2 | CATATTAAGTATATAATATTATATGTGTAGCATATTTCTTGTTTCAGAGCTATGCTGGAA |
| P10 | PFRSM22_gRNA2_N20 | ATATGTGTAGCATATTTCTT |
| P11 | PMG75seqF | CTTTAAATTCATGCAAAAATTTAC |
| P12 | BBHA REV | TGGGCCCGAATTCTCATCATTGTGC |
| P13 | PFMRPL23-5FOUT | GAGCTTTGTTCGATATAC |
| P14 | PFMRPL23-5HRFWD | CTGGTTACCTAGATATCAACCGCGGATATGAACATGAAGTACCGGATAG |
| P15 | PFMRPL23-5HRREV | ATGTCGACCTGCAGTGAAGGTGGTTTCCACTTTGAAGAAGATGACTTCTTGATATTTTTATTTTCACTTGAATATTCAATTGTTTC |
| P16 | PFMRPL23-3HRFWD | ATCCGCGGTCTTAAGGCTTATTTTGTACATATTAG |
| P17 | PFMRPL23-3HRREV | CTGGTTACCATCAGAATTAAATATATACACATTC |
| P18 | PFMRPL23-3FOUT | CGCACATTCTAGTTTGATATTTTG |
| P19 | PFMRPL23_gRNA1 | CATATTAAGTATATAATATTGAAAAATCGTCAAGCAGTAAAGTTTTAGAGCTAGAAATAGC |
| P20 | PFMRPL23_gRNA1_N21 | AAAAATCGTCAAGCAGTAAAG |
| P21 | PFMRPL23_gRNA2 | CATATTAAGTATATAATATTGATTTTTAATTATGTACTATAGTTTTAGAGCTAGAAATAGC |
| P22 | PFMRPL23_gRNA2_N20 | ATTTTTAATTATGTACTATA |
| P23 | N20 CHECK REV | ATATGAATTACAAATATTGCATAAAGA |
|  |  |  |
|  | **RT-qPCR primers** | **Sequence** |
|  | mt rRNA 5 F | ATAGTTACCATAGCTGTAGATG |
|  | mt rRNA 5 R | GTTTTTGGCGGCTGAGCATGT |
|  | mt rRNA 8 F | CTCTACAAAGTTGAACATAGGCTGAGTC |
|  | mt rRNA 8 R | AACTTCTTATAAATGGAAGCGCCGG |
|  | mt rRNA 10 F | TATGTCCTGTTTCAAATATATAT |
|  | mt rRNA 10 R | TTTGATAGCGGTTAACCTTTCC |
|  | mt LSUA F | TTATAGCCATGTCTCCATGAACTAT |
|  | mt LSUA R | ATGATATATCTTCCAAATAGA |
|  | PfGAPDH F | TCCTTGGGGAAAATGCCAAGT |
|  | PfGAPDH R | TGGGGTGTCATCCTTTGGTG |

**References:**

1. Ke, H., et al., The mitochondrial ribosomal protein L13 is critical for the structural and functional integrity of the mitochondrion in Plasmodium falciparum. J Biol Chem, 2018. **293**(21): p. 8128-8137. doi: 10.1074/jbc.RA118.002552.

2. Ke, H., et al., Mitochondrial type II NADH dehydrogenase of Plasmodium falciparum (PfNDH2) is dispensable in the asexual blood stages. PLoS One, 2019. **14**(4): p. e0214023. doi: 10.1371/journal.pone.0214023.

3. Gillespie, D.E., et al., The fragmented mitochondrial ribosomal RNAs of Plasmodium falciparum have short A tails. Nucleic Acids Res, 1999. **27**(11): p. 2416-22. doi: 10.1093/nar/27.11.2416.

4. Boucher, M.J., et al., Integrative proteomics and bioinformatic prediction enable a high-confidence apicoplast proteome in malaria parasites. PLoS Biol, 2018. **16**(9): p. e2005895. doi: 10.1371/journal.pbio.2005895.
