# Supplementary figures and images for: Transcriptional changes in Plasmodium falciparum upon conditional knock down of mitochondrial ribosomal proteins RSM22 and L23
Source: PLoS One. 2022 Oct 6;17(10):e0274993. doi: 10.1371/journal.pone.0274993 (PMC9536634; doi:10.1371/journal.pone.0274993)

Uncropped Western blot images

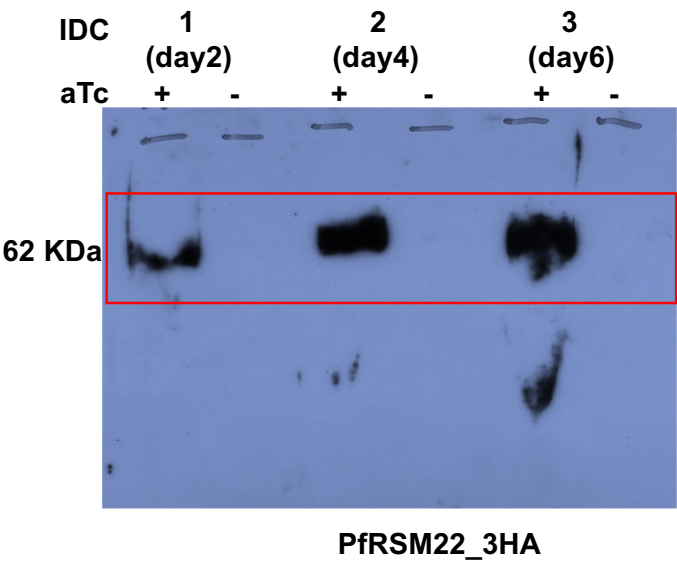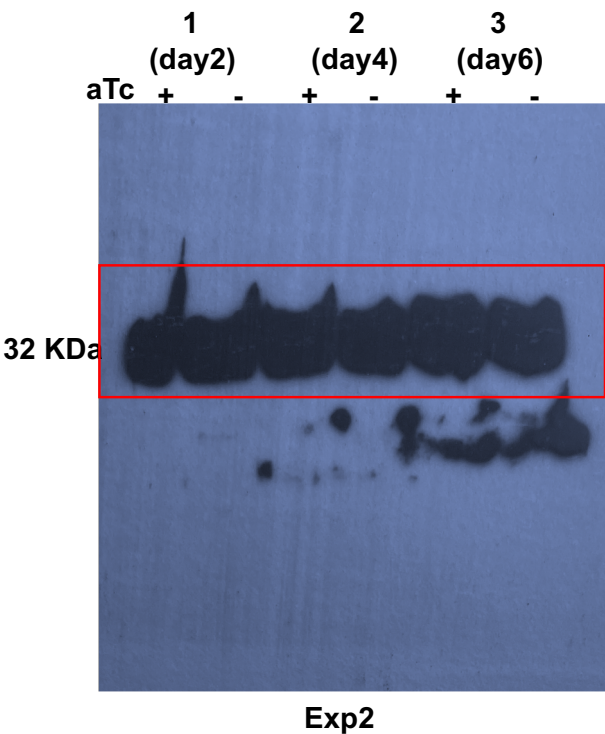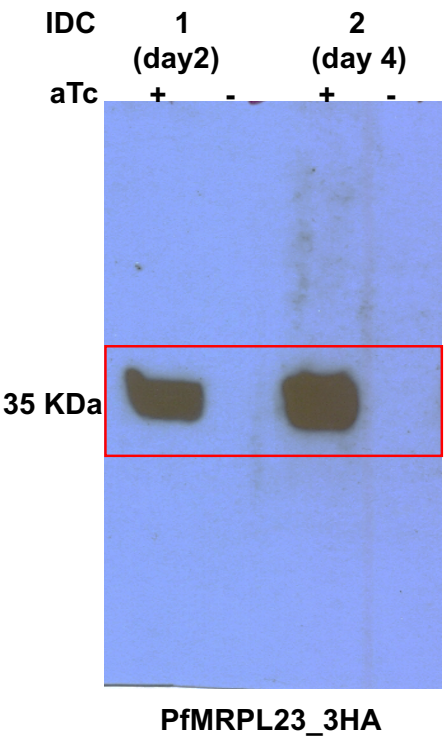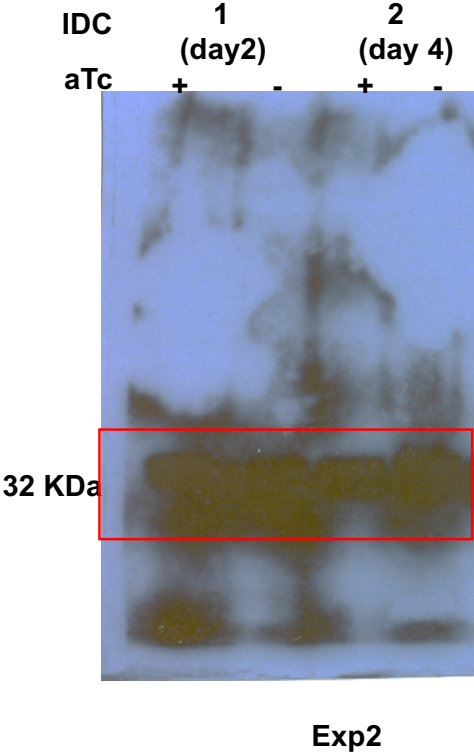

Supplement: S1 Raw images — (PDF) [file pone.0274993.s015.pdf]
