# Supplementary material for: Transcriptional changes in Plasmodium falciparum upon conditional knock down of mitochondrial ribosomal proteins RSM22 and L23
Source: PLoS One. 2022 Oct 6;17(10):e0274993. doi: 10.1371/journal.pone.0274993 (PMC9536634; doi:10.1371/journal.pone.0274993)
Supplement: Attachment — Submitted filename: point-to-point responses to review comments.docx [file pone.0274993.s016.docx]

Review Comments to the Author

Please use the space provided to explain your answers to the questions above. You may also include additional comments for the author, including concerns about dual publication, research ethics, or publication ethics. (Please upload your review as an attachment if it exceeds 20,000 characters)

Reviewer #1: In this manuscript, Dass et al. investigate the function of two putative components of the Plasmodium falciparum mitoribosome. Using conditional knock-downs, the authors convincingly show that both RSM23 and L23 are mitochondrially localized and are important for parasite growth. They then go on to analyze RNA accumulation after 2 days of KD and after 4 and 6 days of KD (the latter only for PfMRPL23 KD). The study is rather descriptive and does not deliver insights into the molecular function of the two putative mitoribosomal proteins.

We thank the reviewer for critical review of our manuscript. While this paper is largely descriptive, we have characterized two new putative mitoribosomal proteins in Plasmodium falciparum that have not been previously studied at all. In this revised version, we provide additional data to support that RSM22 and L23 are mitoribosome related or mitoribosomal proteins. Overall, this paper provides sufficient genetic and biochemical data to reinforce the significance of mitochondrial protein translation and the role of mitoribosomes in malaria parasites.

Major concerns

1. The authors make a number of conclusions based on mitochondrial RNA accumulation. This is problematic since RNA Seq is carried out using polyA-selected RNA. However, it is still an open question in the field, to what extend mitochondrial mRNA and rRNA are polyadenylated and what function polyadenylation has here. This may also be the reason for the failure to detect several known mitochondrial rRNA fragments. Thus, the bias introduced into the RNA seq data of mitochondrial transcripts is potentially huge. This is true if different time points are compared, but is even more relevant if comparisons between different genes are made. All comparisons between the accumulation of rRNA fragments from the small versus the large subunit are difficult. Therefore, it is essential to confirm conclusions on differential expression with orthogonal methods (qRT-PCR; RNA gel blot hybdirization).

We thank the reviewer for this comment. We have used oligoDT probes to enrich transcripts that are polyA positive. Classically, polyA positive transcripts are mRNAs. In our study, we have also found that most mitochondrial (mt) rRNAs are also containing polyA tails. 34 out of 39 mitochondrial rRNA transcripts are detected in our datasets, which suggests that mitochondrial rRNA transcripts are mostly polyA positive and polyA selection is a convenient and efficient way to “capture” them. The three mtDNA encoded mRNA transcripts are expectedly to be polyA positive and detected in our RNAseq. Our data are consistent with the published work done by Feagin et al., which used 3’ RACE and RNAase H analysis to detect polyA tails in many mt rRNA transcripts (PMID 10325433). Since the majority of mtDNA encoded transcripts are present in our datasets, we think our method is not biased. We believe our method is able to determine mitochondrial gene transcription in a large extent. On the other hand, as pointed out by the reviewer, there are 5 mt rRNA transcripts not detected in our study. This could be due to technical issues or a possibility that these 5 transcripts lack polyA tails. Polyadenylation of mt rRNA transcripts is an interesting phenomenon, however, the underlying mechanism is unclear at present. Future investigation is needed to address this intriguing phenomenon.

We agree with the reviewer that validation of RNAseq data with additional methods is necessary. In this revised manuscript, we have performed RT-qPCR to investigate changes of mt rRNA transcription upon knockdown of RSM22 or L23. We chose two mt SSU rRNA genes (RNA 5 and RNA 8) and two mt LSU rRNA genes (RNA 10 and LSU A) since their sizes are relatively big. Most of the highly fragmented mt rRNA genes are very small, which is not suitable for primer design and PCR analysis. As shown in S4 Fig (Supplementary), our RT-qPCR have shown that these mt rRNA genes are differentially expressed in knockdown parasite lines in a trend similar to that shown in RNAseq. These qRT-PCR data support the validity of our RNAseq analysis.

2. Depleting mitoribosomes is expected to lead to massive problems with mitochondrial respiration, which in turn affects the cell on all levels. This obviously includes the accumulation of RNAs in all three genetic compartments. Thus, the RNA pattern observed by RNA seq is the secondary product of a number of problems these KD cells face and is not helpful to understand the primary function of the two proteins investigated. There are some interesting differences in RNA accumulation between the two knock-outs, which might be explained by the different severity of the mutants on mitochondrial translation, but in the end, no insights into the translational capacity of the mutants is shown. A targeted ribosome footprinting analysis or polysome analysis would be needed to understand the impact of the proteins on translation.

We thank the reviewer for this comment. We totally agree with the reviewer that while RNAseq describes the consequences of knocking down RSM22 or L23 on gene transcription, it is unable to directly elucidate the function of these two proteins. Unlike what’s been done in model organisms, however, characterization of mitochondrial protein translation in malaria parasites or other apicomplexan parasites has been extremely difficult due to several technical issues. Firstly, purification of mitochondria in a high purity and quantity from parasites has not been successfully reported in the literature, which poses a challenge to directly assess mitochondrial protein translation with in vitro assays available in model systems. Secondly, since there are only three proteins need to be translated, the number of mitoribosomes per mitochondrion is very low. This is even more problematic for asexual stage malaria parasites, which do not require robust mitochondrial function, e.g., ATP generation, for survival. Mitochondrial respiration in asexual stage parasites is essential, but minimal. Thirdly, there are no antibodies available to detect any of the three proteins translated by mitoribosomes (cytb, COXI, COXIII). Due to these obstacles, we are unable to directly assess the effect on mitochondrial protein translation when RSM22 or L23 is knocked down. Future investigations are needed to solve the forementioned technical issues.

On the other hand, we have developed assays to verify if a protein is related to mitoribosome or mitochondrial protein translation as shown in our published work (PMID: 29626096; PMID: 32273345). The first assay involves growth inhibition assays that measure parasite survivability under chemical inhibition and genetic knockdown towards the same target. If knockdown of RSM22 or L23 affects mitochondrial protein translation, the parasite will become hypersensitive to cyt b inhibitors (atovaquone and ELQ-300) because of synthetic lethality. As a control, the parasite is not expected to change its vulnerability to drugs unrelated to mitochondrial functions such as chloroquine. The second assay directly measures bc1’s enzymatic activity in vitro using crudely purified mitochondria in a spectrometer. Any effect on mitochondrial protein translation will affect cyt b production and, subsequently, bc1 function.

In this revised manuscript, we have provided data to assess bc1 function and parasite’s synthetic lethality in RSM22 and L23 knockdown parasite lines. As shown in Figures 2 and 3, knockdown of RSM22 or L23 leads to reduced bc1 activity, which renders the parasite to become hypersensitive to two bc1 inhibitors, atovaquone and ELQ-300.

3. The two proteins investigated are presumed to be mitoribosome components based on sequence comparisons, but this has not been experimentally shown. In this light, the differential effects on the transcriptome might indicate different functions.

We thank the reviewer for this comment. As explained above, we now provide data to show that RSM22 and L23 are mitoribosomal or mitoribosome related proteins. Our RNAseq analysis has revealed common and differential transcriptomic changes when RSM22 or L23 is knocked down. Although RSM22 and L23 are involved in the same pathway, their time of action on mitochondrial protein translation might be different. Bioinformatics suggest that RSM22 is a mitoribosome assembly factor and L23 is a mitoribosomal protein. Although experimental data are not yet available, we speculate that RSM22 acts at an earlier time point than that of L23 based on studies carried out in other organisms. We agree with the reviewer that our speculation deserves experimental characterization which will be carried out in the future.

4. The idea that PfRSM22 is an assembly factor of the mitoribosome is not substantiated by any data. There is no evidence shown for “mitoribosome synthesis inhibition” (lines 146-148).

We thank the reviewer for this comment. As shown in Fig S1, we have performed extensive bioinformatics analysis to change the annotation of the gene (Pf3D7_102700) from S22 to RSM22. We agree with the reviewer that direct biochemical characterization of PfRSM22 is not yet available. We have mentioned several times in the manuscript that this annotation is putative and RSM22 is likely a mitoribosome assembly factor. Future studies are needed to directly assess the role of PfRSM22 in mitoribosome assembly. We agree with the reviewer that we have not provided direct evidence showing mitoribosomal synthesis inhibition (line 146). In this revised manuscript, “mitoribosome synthesis inhibition in line 146 has been deleted.

Minor concern

The authors do not provide access to the raw data which precludes any analysis on the reviewers’ site. Usually, SRA accessions (or the like) are made available for the reviewing process.

We thank the reviewer for this comment. We are sorry that we missed this during manuscript submission. We have deposited all RNAseq raw data in NCBI. The link is provided as follows.

https://www.ncbi.nlm.nih.gov/sra/PRJNA801275

Reviewer #2: The authors present an article on the study of Plasmodium falciparum, of which two nuclear genes coding for mitochondrial proteins have been KD. Their choices fell on the PfRSM22 and PfMRPL23 genes without any reason being known (continuation of their previous studies ?). The authors used a conditional KD system to explore the transcriptomic bulk response of erythrocyte stages at two stages : early (day 2 post KD) and late (More than 6 days post KD) phases. By comparing the differential expression of other proteins, the authors propose a series of new potentially MRP-like proteins.

The manuscript is well written and very clear. The technical approach is convincing and the results are well presented. They attest to the localization of these two proteins in the mitochondria and also show that the KD of these genes leads to the death of the parasites at 8 and 6 days post KD for PfRSM22 and PfMRLP23 respectively.

We thank the reviewer for critical review of this manuscript. We appreciate the positive comments on our manuscript given by this reviewer.

However, I have two remarks:

The first concerns the methodology adopted in the analysis of the sequencing results. In their materials and methods the authors state that transcriptomic data are analyzed in duplicate using a set of tools including clusterProfiler R Package. The results presented are not exactly comparable with those found using PlasmoDB algorithm from Gene Ontology Enrichment pages using their available data. It would seem relevant to me to discuss the convergences found between these two methodologies and how they could reinforce the robustness of the data presented.

We thank the reviewer for this comment. Our RNAseq was carried out by a commercial source (Novogene) that has its standard data analysis strategy. The regulated KEGG pathways were analyzed by clusterProfiler R package. We thank the reviewer for bringing this analysis method using PlasmoDB tools to our attention. In this revised manuscript, we have now also analyzed the differentially regulated genes found in the RNAseq data with the PlasmoDB GO term analysis. The results are now provided in S3 Table, along with the enriched KEGG pathway results. Some similarities of the processes to KEGG pathways are suggested by the GO term descriptions. All differentially regulated gene IDs are provided in S3 Table, from KEGG analysis or GO enrichment by PlasmoDB tools. As shown in S3 Table, every single significant GO term is related to one or more KEGG pathways. We believe there is significant overlay or convergence between the two analysis methods.

The second remark concerns the relevance of analyzing the transcriptome at the late phase where it seems at least that a majority of the parasites are already dead (figure 1 E) and 1 F)), the data collected are therefore in my opinion difficult if not impossible to analyze and certainly subject to controversy.

We thank the reviewer for this comment. The late phase of RSM22 knockdown refers to day 6 post aTc removal whereas the late phase of L23 knockdown indicates day 4 post aTc removal. As shown in Figs 1E and 1F, on these days (day 6 of RSM22 and day 4 of L23), the parasites were largely healthy. They were able to make new rings on the next day. We therefore included these time points in our RNAseq study to understand transcriptomic changes right before the onset of parasite death. We did not perform the study on day 8 aTc removal for RSM22 or day 6 aTc removal for L23 because parasites were largely dead (Figs 1E and 1F). We agree with the reviewer that analysis of dead parasites is problematic. That is why we chose to analyze transcriptome when parasites were relatively healthy.
